# 2503 First year medical student characteristics associated with readiness to talk about race

**DOI:** 10.1017/cts.2018.211

**Published:** 2018-11-21

**Authors:** Brooke Cunningham, Rachel Hardeman, Samantha Carlson

**Affiliations:** 1 Health Policy and Management, University of Minnesota School of Public Health, Minneapolis, MN, USA; 2 Professional Data Analysts, Minneapolis, MN, USA

## Abstract

OBJECTIVES/SPECIFIC AIMS: Calls to break the silence around the effects of racism on health are growing. Few researchers have examined the relationship between medical student characteristics and students’ comfort, motivation, and skill to discuss racism. This paper examines medical student characteristics associated with readiness to talk about racism among first-year medical students at the University of Minnesota. METHODS/STUDY POPULATION: In February 2017 prior to a lecture on racism and health, we invited first year medical students to participate in a web-based survey about their experiences and comfort discussing racism. We calculated descriptive statistics and measured differences by student race (White vs. Asian vs. Black/multiracial/other) and undergraduate major type (STEM vs. non-STEM) using χ^2^ tests for variables with categorical responses and generalized linear regression models with pairwise comparisons (i.e., 2-sample *t*-tests) for variables with continuous responses. RESULTS/ANTICIPATED RESULTS: (n=107/163). The majority of students were male (53%); White (75%); and majored in STEM majors in college (85%). College major was not associated with race. Students’ responses to multiple items suggest that the vast majority perceived racial inequality as a major problem in the United States. Race was significantly associated with only 1 of these items. Specifically, 100% (16/16) of Black/multiracial/other students [under-represented minority (URM) students] reported “too little attention” is paid to race and racial issues, while only 53% of White students (42/79) and 55% of Asian students (6/11) chose this response. Students with non-STEM majors and students who identified as URM students reported talking about racism with friends more often than STEM majors and white students, respectively. In conversations about race at school, two-thirds of students were concerned that they might unintentionally offend others or be misunderstood. However, non-STEM majors and URM students were significantly less worried that they would unintentionally offend others in conversations about race at school than STEM majors and white students. Larger percentages of URM students (50%) than White students (25%) were afraid that others would not respect their views because of their race. White students were more afraid that they might that they would be called racist than URM students. DISCUSSION/SIGNIFICANCE OF IMPACT: Many students find it challenging to discuss race and racism in medical education settings. URM students and non-STEM majors reported greater frequency talking about racism with friends and appear to be less anxious in conversations about racism than White students and STEM majors respectively. Given non-STEM majors' greater psychological safety discussing racism, future research should explore whether non-STEM majors are better prepared and more motivated to address racial disparities in health and health care than STEM majors. Such research could have important implications for medical school admissions.

